# Fumaric Acids Directly Influence Gene Expression of Neuroprotective Factors in Rodent Microglia

**DOI:** 10.3390/ijms20020325

**Published:** 2019-01-15

**Authors:** Jessica Kronenberg, Kaweh Pars, Marina Brieskorn, Chittappen K. Prajeeth, Sandra Heckers, Philipp Schwenkenbecher, Thomas Skripuletz, Refik Pul, Andreas Pavlou, Martin Stangel

**Affiliations:** 1 Clinical Neuroimmunology and Neurochemistry, Department of Neurology, Hannover Medical School, 30559 Hannover, Germany; kronenberg.jessica@mh-hannover.de (J.K.); Kaweh.pars@uni-oldenburg.de (K.P.); Brieskorn.marina@mh-hannover.de (M.B.); ChittappenKandiyil.Prajeeth@mh-hannover.de (C.K.P.); sandra.heckers@gmx.de (S.H.); Schwenkenbecher.philipp@mh-hannover.de (P.S.); skripuletz.thomas@mh-hannover.de (T.S.); refik.pul@uk-essen.de (R.P.); pavlou.andreas@mh-hannover.de (A.P.); 2 Center for Systems Neuroscience, University of Veterinary Medicine Hannover, 30559 Hannover, Germany; 3 Department of Neurology, European Medical School, University Oldenburg, 26129 Oldenburg, Germany; 4 Department of Neurology, University Clinic Essen, 45147 Essen, Germany

**Keywords:** microglia, dimethylfumarate, monomethylfumarate, IGF-1

## Abstract

Dimethylfumarate (DMF) has been approved the for treatment of relapsing-remitting multiple sclerosis. The mode of action of DMF and its assumed active primary metabolite monomethylfumarate (MMF) is still not fully understood, notably for brain resident cells. Therefore we investigated potential direct effects of DMF and MMF on microglia and indirect effects on oligodendrocytes. Primary rat microglia were differentiated into M1-like, M2-like and M0 phenotypes and treated in vitro with DMF or MMF. The gene expression of pro-inflammatory and anti-inflammatory factors such as growth factors (IGF-1), interleukins (IL-10, IL-1β), chemokines (CCl3, CXCL-10) as well as cytokines (TGF-1β, TNFα), iNOS, and the mannose receptor (MRC1) was examined by determining their transcription level with qPCR, and on the protein level by ELISA and FACS analysis. Furthermore, microglia function was determined by phagocytosis assays and indirect effects on oligodendroglial proliferation and differentiation. DMF treatment of M0 and M1-like polarized microglia demonstrated an upregulation of gene expression for IGF-1 and MRC1, but not on the protein level. While the phagocytic activity remained unchanged, DMF and MMF treated microglia supernatants led to an enhanced proliferation of oligodendrocyte precursor cells (OPC). These results suggest that DMF has anti-inflammatory effects on microglia which may result in enhanced proliferation of OPC.

## 1. Introduction

Multiple sclerosis (MS) is the most common chronic disease of the central nervous system (CNS) in young adults [1]. It is characterized by inflammation, de- and remyelination and neuronal degeneration [2,3]. There are different cells postulated to be involved in these processes. One of them is microglia, brain-resident immune cells [4]. Microglia can interact with antigens and serve as antigen-presenting cells. Phagocytosis is a hallmark of microglia and due to different effector molecules microglia are thought to be involved the in development of CNS lesions as well as in neuroprotection and remyelination [5,6]. Under environmental influence microglia have the possibility to react in different ways by changing into different phenotypes that respond as pro-inflammatory M1-like microglia, releasing pro-inflammatory cytokines [7], and as an anti-inflammatory M2-like phenotype [8,9]. During inflammation the cells are rapidly activated and several factors essential for remyelination are produced by microglia [10]. One of these factors is insulin-like growth factor-1 (IGF-I), which has been demonstrated to have neurotrophic effects on neural tissue, notably promoting oligodendrocyte differentiation in vitro [11,12,13,14]. As microglia may have a neuroprotective influence, a major therapeutic challenge is to modulate microglia cells to exert their beneficial properties.

Dimethylfumarate (DMF) is an approved drug for the treatment of relapsing-remitting MS [15]. Although the mode of action of DMF and its active metabolite monomethylfumarate (MMF) [16] is not yet fully understood some investigations showed an effect through the activation of the Nrf2 transcriptional pathway which leads to enhanced cytoprotection, decreased oxidative stress, and reduced neuroinflammation [17,18,19]. Several in vitro studies demonstrated that DMF has immune-modulatory properties on T cells by promoting their cytokine release profile towards anti-inflammatory Th2 cells rather than proinflammatory Th1 and Th17 cells [20,21], and might modulate microglia activation and their phenotype [22,23]. In order to elucidate the effects of DMF on microglia, we first analyzed modulatory influences of DMF and MMF on regulation of cytokines and growth factors in different microglial phenotypes in vitro and analyzed secondary effects of DMF and MMF on proliferation and differentiation of oligodendrocyte precursor cells.

## 2. Results

### 2.1. Phagocytic Activity is Unaffected by DMF and MMF

**Figure 1:**
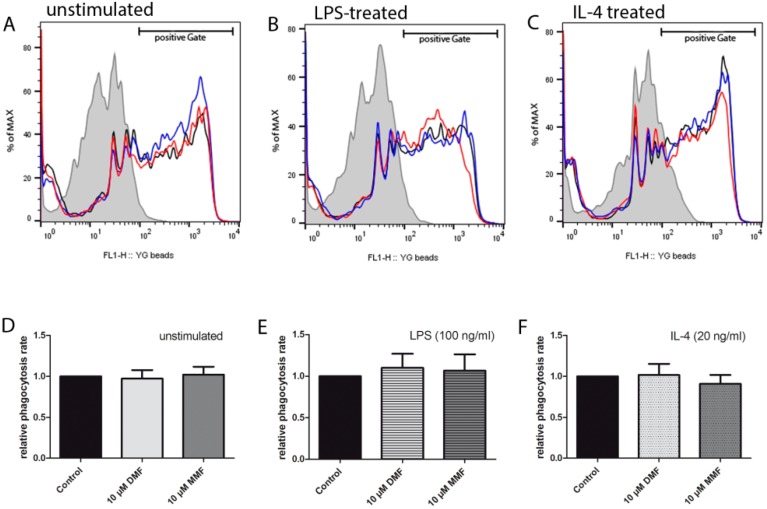
Phagocytic activity of microglia is unaffected by DMF and MMF. Neither DMF nor MMF changed the phagocytic activity of microglia. Flow cytometric histograms of unstimulated (**A**), LPS treated (**B**), and IL-4 treated microglia (**C**). The mean fluorescence intensity reflects the amount of phagocytosed fluorescent latex beads (**D**–**F**) (filled: 4 °C, black line: control, red line: 10 µM DMF, blue line: 10 µM MMF). Data is shown as the ratio of untreated microglia in relation to DMF- or MMF-treated microglia. All data are presented as the arithmetic means ± SEM of *n* = 5.

One of the major functions of microglia during inflammation is phagocytosis of cell debris. Microglia can be classified in functionally pro-inflammatory (M1-like) and anti-inflammatory (M2-like) phenotypes [6,24]. Microglia can be stimulated by LPS and shift into an M1-like phenotype that expresses pro-inflammatory cytokines. Stimulation of microglia by IL-4 can lead in to a shift into an M2-like phenotype [10]. Therefore, we investigated the effects of DMF and MMF on primary microglia (M0), M1-like, and M2-like microglia. After 24 h of treatment with DMF or MMF, microglia were stimulated with LPS or IL-4 for 6 h to induce an M1- or M2-like phenotype, respectively. Phagocytic activity was measured by uptake of latex beads with flow cytometry. As demonstrated in Figure 1, neither DMF nor MMF affected the phagocytic activity of untreated, LPS treated or IL-4 treated microglia.

### 2.2. Effects of DMF and MMF on Gene Expression in Microglia

**Figure 2:**
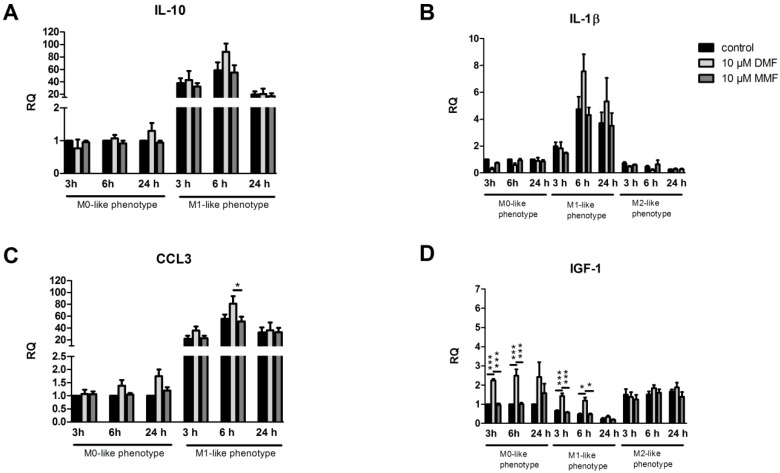

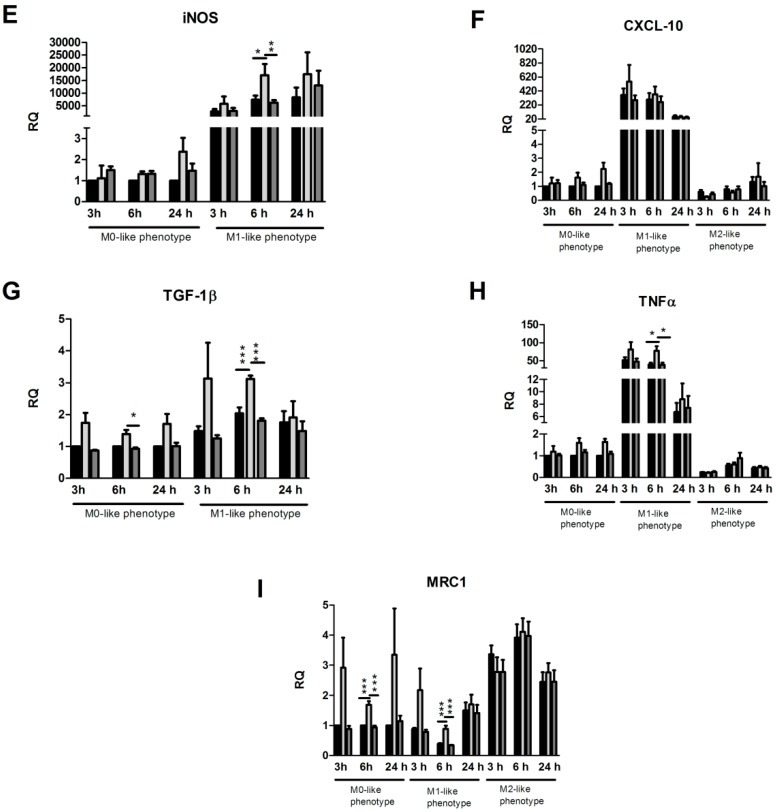
Effects of DMF and MMF on gene expression in microglia. Microglia were treated with medium, 10 µM DMF or 10 µM MMF for 24 h and afterwards stimulated with LPS (100 ng/mL) or IL-4 (20 ng/mL) for another 3, 6 or 24 h. Graphs show mRNA expression fold changes of IL-10 (**A**), IL-1β (**B**), CCL3 (**C**), IGF-1 (**D**), iNOS (**E**), CXCL-10 (**F**), TGF1-β (**G**), TNFα (**H**) and MRC1 (**I**), calculated for untreated and unstimulated cells, normalized with HPRT-1 and using the ΔΔCT method. Data are presented as the arithmetic means ± SEM of *n* = 4–6. Significant differences are marked by asterisks (* *p* < 0.05; ** *p* < 0.01; *** *p* < 0.001).

After treatment of primary microglia (M0) with DMF (10 µM) or MMF (10 µM) for 24 h, cells were either kept in control medium or stimulated with LPS or IL-4. We then determined the gene expression of cytokines and growth factors compared to untreated control cells after 3 h, 6 h and 24 h, respectively. As demonstrated in Figure 2, pro-inflammatory factors such as TNFα, IL-1β, and iNOS were upregulated after LPS stimulation whereas anti-inflammatory factors such as IGF-1 and MRC1 were upregulated after IL-4 stimulation. Treatment of microglia with DMF or MMF prior to stimulation with IL-4 had no effects on any gene expression. Microglia treated with DMF showed a significant upregulation in IGF-1 expression in M0-like and M1-like microglia after 3 h and 6 h. Furthermore, iNOS, TGF-1β and TNFα were significantly upregulated in microglia after DMF treatment and 6 h of LPS stimulation. MRC1 showed a significant upregulation in M0-like and M1-like microglia after treatment with DMF for 24 h and stimulation for 6 h. The expression of interleukins IL-10 and IL-1β as well as the expression of chemokines CCL-3 and CXCL-10 remained unchanged after DMF treatment.

### 2.3. Effects of DMF on IGF-1 Protein Expression

**Figure 3:**
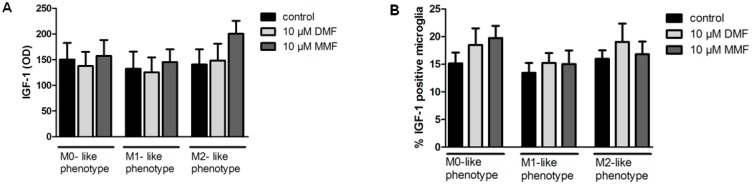
Effects of DMF and MMF on IGF-1 expression. Secretion of IGF-1 protein by microglia was assessed by ELISA. Microglia were treated with medium, 10 µM DMF or 10 µM MMF for 24 h and stimulated with LPS or IL-4 for 6 h. Cell culture supernatants were analyzed for IGF-1 secretion by ELISA (**A**). Cell pellets of the same experimental setting were analyzed for IGF-1 production with FACS analysis (**B**) Data are presented as the arithmetic means ± SEM of *n* = 4.

As we demonstrated that IGF-1 gene expression is upregulated in M0- and M1-like microglia treated with DMF, we performed further ELISA and FACS analysis of IGF-1 in rat microglia. Cell cultures were treated with DMF or MMF for 24 h and stimulated with LPS or IL-4 for another 6 h. Supernatants were analyzed by ELISA and cells by FACS analysis. Surprisingly, IGF-1 protein levels were not upregulated in microglial supernatants or in cells (Figure 3) and did thus not correlate with gene expression after DMF or MMF treatment, respectively.

### 2.4. Supernatants from Microglia Treated with DMF or MMF Enhance Oligodendrocyte Precursor Proliferation

In order to investigate functional consequences of the effect of DMF and MMF on microglia, we investigated whether microglia supernatants enhanced differentiation or proliferation of oligodendrocyte precursor cells (OPCs). Primary rat OPCs were plated for 24 h in either differentiation or proliferation medium alone and afterwards incubated with microglia supernatants derived from polarized microglia treated with DMF (10 µM) or MMF (10 µM), for a further 48 h. To investigate whether IGF-1 alone influenced oligodendrocytes, the cells were incubated with 100 ng/mL IGF-1 in culture medium for 48 h. Incubation of OPC with M1-like microglia supernatant was toxic for OPCs and strongly diminished the absolute number of oligodendrocytes, thus differentiation and proliferation could not be calculated.

**Figure 4:**
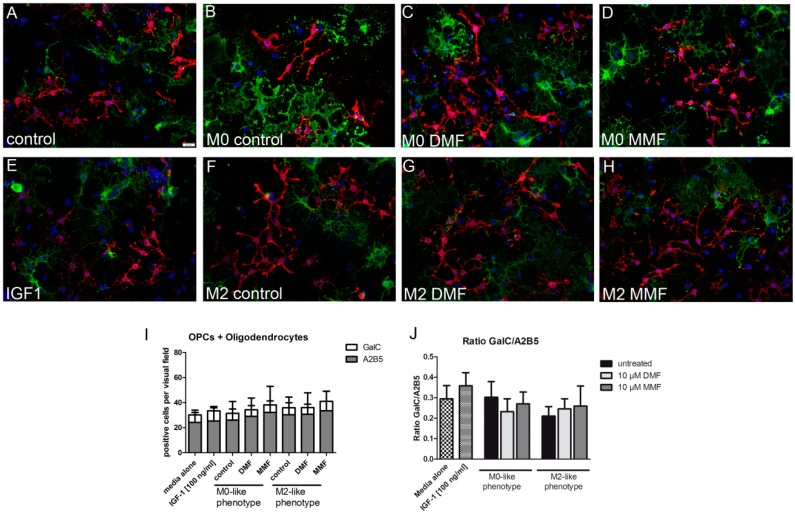
Effect of microglia supernatants treated with DMF or MMF on oligodendrocyte differentiation. OPCs were plated in differentiation medium for 24 h and were incubated for further 48 h with supernatants from polarized treated with medium, 10 µM DMF or 10 µM MMF. A2B5 (*red*)/GalC (*green*) double staining of cell cultures (**A**–**H**). There was no change in the absolute number of A2B5 or GalC positive cells (**I**). The differentiation index is presented as the ratio of GalC/A2B5 positive cells remained unchanged (**J**). Data are presented as arithmetic means ± SEM of *n* = 5–6.

**Figure 5:**
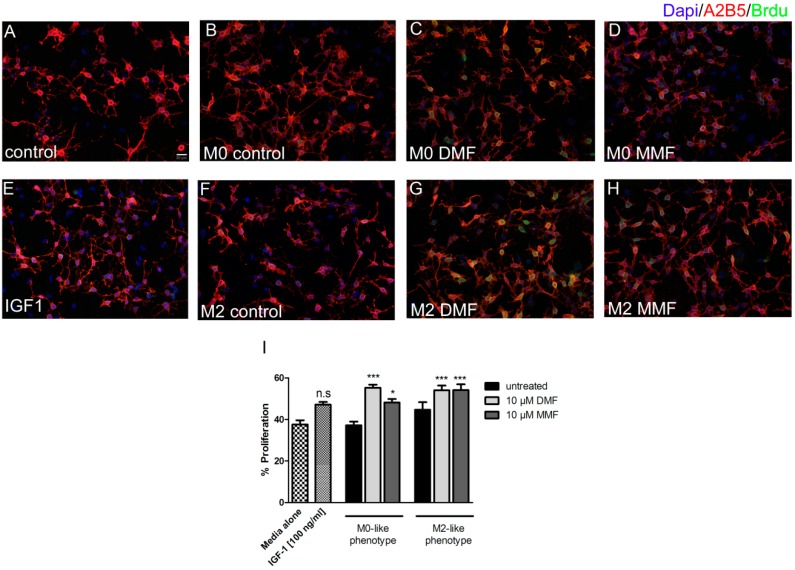
Effect of microglia supernatants treated with DMF or MMF on OPC proliferation. OPCs were plated in proliferation medium for 24 h and were incubated for further 48 h with supernatants from polarized microglia treated with medium, 10 µM DMF or 10 µM MMF. A2B5 (*red*)/BrdU (*green*) double staining of cell cultures (**A**–**H**). Proliferation of OPC was increased after incubation with M0-like and M2-like microglia supernatants, which were treated with 10 µM DMF or 10 µM MMF (**I**). Data are presented as arithmetic means ± SEM of *n* = 4. Significant differences are marked by asterisks (* *p* < 0.05; ** *p* < 0.01; *** *p* < 0.001).

A2B5 (a marker for oligodendrocyte precursor cells) and GalC (a marker for mature oligodendrocytes) double staining was performed to determine the differentiation index of oligodendrocytes. Following a 48 h treatment with supernatants of DMF, MMF treated microglia or IGF-1 alone, the amount of mature oligodendrocytes and OPCs were not affected. Similary, the differentiation index for GalC/A2B5 remained unchanged (Figure 4). Proliferation of oligodendrocytes was determined by BrdU and A2B5 double staining. Treatment for 48 h with control M0-like, M2-like microglia supernatants or IGF-1 alone did not alter the proliferation of oligodendrocytes. However, proliferation was significantly enhanced after incubation with supernatants from DMF or MMF treated M0-like and M2-like microglia (Figure 5). In contrast, differentiation of OPCs into mature oligodendrocytes was not affected by the supernatants from DMF or MMF treated microglia.

## 3. Discussion

In this study we could identify several effects of DMF on microglia in vitro. On the gene expression level, our results suggest that DMF directly shifts microglia into a rather anti-inflammatory reaction even under inflammatory conditions. An indirect effect of fumaric acids on OPCs is the enhancement of OPC proliferation. However, we consider that further in vivo investigations are required to elucidate the relevance of these effects for therapeutic strategies in MS.

It is well described that fumaric acids have a positive influence on the immune system and DMF has been approved for the treatment of relapsing-remitting MS [15]. In several investigations a neuroprotective effect of DMF, and its assumed clinically relevant metabolite MMF, is postulated [3,4,5,6]. Recently, Kornberg et al. reported an effect of DMF and MMF on GAPDH, and thereby the downregulation of aerobic glycolysis in activated myeloid and lymphoid cells [25]. Although the concept of interference with the metabolism seems to be a reasonable target for fumaric acids on peripheral cells, the authors underline that the precise mode of actions of DMF and MMF still remains uncertain. In a complex interplay there are different cells involved in the autoimmune process of MS. As brain resident cells, microglia play an important role in inflammatory and anti-inflammatory processes. A hallmark of microglia is the ability of phagocytosis. Additionally, depending on the environmental influence, microglia can shift into a pro- or anti-inflammatory phenotype and perpetuate these processes by cytokine production. We therefore investigated the influence of DMF and MMF on non-stimulated microglia (M0), pro-inflammatory microglia (M1-like), anti-inflammatory microglia (M2-like), and a possible secondary influence on OPCs. We could demonstrate that DMF and MMF have no influence on the phagocytosis of microglia without any stimulation (M0), under LPS stimulation (M1-like), or under IL-4 stimulation (M2-like). To investigate a possible influence of DMF or MMF on shifting into a certain phenotype, we treated microglia with DMF or MMF and then stimulated with LPS or IL-4, respectively. As expected, gene expression of pro-inflammatory marker after LPS stimulation (TNFα, IL-1β and iNOS) and anti-inflammatory marker after IL-4 stimulation (IGF-1 and MRC1) were upregulated in comparison to unstimulated M0 cells. DMF or MMF had no influence on the gene expression of cytokines after IL-4 treatment of microglia. Similarly, MMF treatment did not influence any gene expression after LPS stimulation. However, treatment of microglia with DMF led to a small but significant upregulation of iNOS, TGF-1β, TNFα, and CCL3 after 6 h of LPS stimulation. Furthermore, in DMF treated M0 and M1-like microglia, there was a significant upregulation of gene expression of MRC1 (6 h) and IGF-1 (3 h, 6 h). We therefore postulate that DMF may induce a shift of M0 microglia towards an anti-inflammatory phenotype in an inflammatory environment. IGF-1 is a growth factor that in vitro demonstrates neurotrophic effects and promotes oligodendrocyte differentiation [13,14]. We therefore explored an upregulation of IGF-1 on the protein level in microglia supernatants via ELISA and in microglia cells via FACS analysis. Interestingly, in both ELISA and FACS analysis, we were not able to detect a significant protein upregulation. A discrepancy in gene and protein level regulation has been reported before, and there are several publications discussing the quantification of gene expression [26,27]. Protein levels correlate with only 30–40% of their corresponding mRNA abundances, and some combination of post-transcriptional regulation and measurement noises are discussed as possible reasons of that poor correlation. Furthermore, the role of other translation regulators (like RNA-binding proteins) or the kinetics of translational process are still unclear [28]. It can only be speculated which of these mechanisms is to be made responsible in our culture system. Regarding the important role of oligodendrocytes in de- and re-myelination in MS [29], we nevertheless studied the influence of DMF or MMF treated microglia on OPCs. Interestingly, IGF-1 alone did not show any influence on proliferation or differentiation of OPCs. However, we could demonstrate a significantly enhanced proliferation of OPCs in the presence of supernatants from DMF and MMF treated M0 and M2-like microglia. Incubation with supernatants of M1-like microglia seemed toxic and diminished the number of OPCs strongly, so differentiation and proliferation could not be calculated. Supernatants from M0 and M2-like microglia had no effect on the differentiation of OPCs into mature oligodendrocytes. Since IGF-1 does not seem to be responsible for the enhanced proliferation of OPCs treated with supernatants from DMF and MMF treated M0 and M2-like microglia, there must be other yet unidentified factors secreted by these microglia that mediate this effect. Further studies including appropriate animal models are necessary to dissect these mechanisms in detail.

## 4. Materials and Methods

### 4.1. Mixed Glia Cell Cultures

For preparation of primary mixed glial cell cultures, neonatal Sprague-Dawley rats P0-P3 were used. As previously described [30] brains were freed from the meninges, choroid plexus and brain stem. Afterwards they were dissociated mechanically and further enzymatically digested with 0.1% trypsin (Biochrom, Berlin, Germany) and 0.25% DNase (Roche, Mannheim, Germany). The single cell suspension was then plated into culture flasks pre-coated with poly-l-lysine (PLL; Sigma-Aldrich, Hamburg, Germany). Cultures were kept in Dulbecco’s Modified Eagle Medium (DMEM; Thermo Fisher Scientific, Waltham, MA, USA) supplemented with 1% penicillin/streptomycin (Sigma-Aldrich, Saint Louis, MO, USA) and 10% fetal bovine serum (FBS; Biochrom, Berlin, Germany) at 37 °C and 5% CO_2_ until use.

On day 7, microglial cells were isolated by shaking at 37 °C for 45 min at 180 rpm on an orbital shaker (Edmund Bühler, Heching, Germany) and afterwards 500,000 cells were seeded into 6 well plates (Sarstedt, Nümbrecht, Germany). Microglia were incubated overnight at 37 °C, with 5% CO_2_ and on the following day treated with 10 µM DMF or 10 µM MMF for 24 h, followed by stimulation with LPS (100 ng/mL; Sigma-Aldrich, Hamburg, Germany) or IL-4 (20 ng/mL; Peprotech, Hamburg, Germany) for either 3 h, 6 h or 24 h. 

After resting for at least 2 h, oligodendrocytes were isolated from the remaining culture flasks by shaking at 180 rpm for 16–20 h. Supernatants were collected, centrifuged and cells were then transferred into an uncoated flask for 30 min at 37 °C to reduce contamination with astrocytes and microglia. Approximately 60,000 cells were plated on poly-l-lysine (PLL) coated 12 mm glass coverslips and cultured in proliferation or differentiation medium for 24 h, followed by incubation for another 48 h with DMF or MMF treated microglia supernatant. Differentiation medium consisted of Neurobasal^®^ medium supplemented with GlutaMAX™, B-27 supplement (all from Thermo Fisher Scientific, Osterode, Germany), and 30 ng/mL T3 (Sigma-Aldrich, Hamburg, Germany) and proliferation medium consisted of KnockOut™ DMEM/F-12 supplemented with GlutaMAX™, StemPro supplement, EGF, human FGF, PDGF-AA (all from Thermo Fisher Scientific, Osterode, Germany).

### 4.2. RNA Isolation and Reverse Transcription Polymerase Chain Reaction (RT-PCR)

Real-time quantitative polymerase chain reaction (qPCR) was performed for the genes IL-1β, tumor necrosis factor-α (TNFα), chemokine (C-X-C motif) ligand 10 (CXCL10), mannose receptor 1 (MRC1), insulin-like growth factor-1 (IGF-1), arginase 1 (Arg1), Chemokine (C-C motif) ligand 3 (CCL3), and inducible nitric oxide synthase (iNOS). Extraction of ribonucleic acid (RNA) was done by using the RNeasy Micro Kit (Qiagen, Hilden, Germany) according to the manufacturer’s instructions. RNA concentration was measured with a NanoDrop 2000 spectrophotometer (Thermo Fisher Scientific, MA, USA). Complementary deoxyribonucleic acid (cDNA) was synthesized using the High Capacity cDNA Reverse Transcription Kit (Applied Biosystems, Foster City, CA, USA) and qPCR analyses were performed with the StepOneTM Real-Time PCR System and TaqMan assays (Applied Biosystems, Foster City, CA, USA) (Table 1). The ΔΔCT method was used to determine differences in the expression between untreated control and treated primary cells. Multiple comparisons for the housekeeping genes hypoxanthine-guanine-phosphoribosyl-transferase 1 (HPRT-1) and Glycerinaldehyd-3-phosphat-Dehydrogenase (GAPDH) were used for gene normalization.

### 4.3. Phagocytosis Assay

Phagocytic activity of microglia was determined by measuring the uptake of fluorescent latex beads on flow cytometry as previously described [31]. After treatment with DMF or MMF for 24 h, followed by stimulation with IL-4 or LPS for another 6 h, microglia were incubated with latex beads (1 μm, Fluoresbrite™ Yellow Green carboxylate microspheres; Polysciences, Warrington, PA, USA) in a cell/bead ratio of 1:100 for 1 h at 37 °C. As a negative control, the same condition was incubated at 4 °C. To remove non-internalized beads, cells were washed several times with ice-cold PBS and harvested in 0.1% trypsin/EDTA solution. The uptake of latex beads was then analyzed by flow cytometry (FACSCalibur; Becton-Dickinson, San Jose, CA, USA). To assess phagocytosis, a shift in mean fluorescence intensity (MFI) resulting from the uptake of fluorescent beads and the percentage of gated microglia that phagocytosed latex beads, were used. Active phagocytosis was rated by computing measured values of microglia incubated at 4 °C and the values accessed at 37 °C.

### 4.4. Enzyme-linked Immunosorbent Assay (ELISA)

Free IGF-1 concentrations in the cell supernatants were determined using the IGF-1 ELISA Kit (R&D System, Wiesbaden, Germany) according to the manufacturer’s instructions. Supernatants were harvested from microglia treated with DMF and MMF for 24 h followed by stimulation with IL-4 or LPS for another 6 h. 50 µL of standard, control, or sample were added per well and incubated for 2 h at room temperature on a horizontal shaker. After washing, IGF-1 conjugate was added to each well and incubated for another 2 h. Following the addition of substrate solution, the enzyme reaction color product was calculated by subtracting wavelengths detection at 570 nm from the readings at 450 nm with Sunrise-Basic Tecan (Tecan, Grödig, Austria). 

### 4.5. Flow Cytometry of IGF-1

Microglial production of IGF-1 was determined by flow cytometry. Cells were treated with DMF and MMF for 24 h followed by stimulation with IL-4 or LPS for another 6 h. For the last 4 h, Brefeldin A (10 mg/ml; Sigma-Aldrich, Hamburg, Germany) was added to the culture. After fixation and permeabilization (eBioscience, San Diego, CA, USA), microglia were stained for IGF-1 1:500 (Abcam, Cambridge, UK) for 30 min at room temperature. After washing with phosphate-buffered saline (PBS) cells were incubated with the secondary antibody AlexaFluor 488 goat anti-rabbit IgG (Thermo Fisher Scientific, Osterode, Germany). All measurements were performed in triplicates per condition and in four independent experiments. The analyses were performed using the FACSCalibur platform with FlowJow Software (BD Beckston Dickinson, Franklin Lakes, NJ, USA).

### 4.6. Oligodendrocytes Assays

Two different approaches were performed to investigate the effect of microglia supernatants on oligodendrocytes. For this, microglia were pre-treated with DMF or MMF for 24 h and further stimulated with LPS or IL-4 for 6 h in serum-free culture medium. Supernatants were harvested and stored at −80 °C until further use. Oligodendrocytes were plated and allowed to proliferate or differentiate in normal culture medium for 24 h and were then incubated at a ratio of 1:3 with defined culture media/microglia supernatants for 48 h.

To determine the differentiation index of mature oligodendrocytes to OPCs, primary cells were incubated with anti-A2B5 (hybridoma supernatant, clone 105, European Collection of Cell Cultures) and anti-galactocereboside (GalC, hybridomasupernatant, clone IC-07, European Collection of Cell Cultures) antibodies for 30 min at 37 °C. After fixation with 4% paraformaldehyde (PFA), cells were incubated with secondary antibodies AlexaFluor 488 goat anti-mouse IgG3 and AlexaFluor 555 goat anti-mouse IgMμ 1:500 (Thermo Fisher Scientific, Osterode, Germany).

To investigate the percentage of proliferating oligodendrocytes, cells were incubated for 3 h with 10 µM Bromodeoxyuridine (BrdU, Roche, Indianapolis, IN, USA). Cultures were washed with PBS and incubated with anti-A2B5 supernatant. After fixation with 4% PFA, cells were permeabilized with methanol at −20 °C and DNA was denatured with hydrochloric acid (2M HCl, Roth, Karlsruhe, Germany). Cells were then neutralized with 0. 1 mol/lborate buffer pH 8.5, stained with anti-BrdU 1:100 (Roche, Indianapolis, IN, USA) and incubated with secondary antibodies AlexaFluor 555 goat anti-mouse IgMμ and AlexaFluor 488 goat anti-mouse IgG 1:500 (Thermo Fisher Scientific).

### 4.7. Statistical Analysis

All experiments were performed at least four times in independent experiments. GraphPad Prism version 5.02 was used for statistical analysis (GraphPad Software, Inc., La Jolla, CA, USA). One-way ANOVA followed by the Tukey’s Multiple Comparison Test was used for gene expression and Kruskal-Wallis test followed by the Dunn’s Multiple Comparison Test was used for all other statistical analyses. Values are presented as arithmetic means ± standard error of the mean (SEM). *p* < 0.05 was considered to indicate a statistically significant difference.

## Figures and Tables

**Table 1:** Primer used for polymerase chain reaction.

Gene	Gene Expression Assay Number
CCL3	Rn_01464736_g1
TGF-1β	Rn_00572010_m1
Il-10	Rn_00563409_m1
MRC-1	Rn_01487342_m1
CXCL-10	Rn_01413889_m1
Il-1ß	Rn_00580432_m1
IGF-1	Rn_00710306_m1
TNFα	Rn_99999017_m1
iNOS	Rn_00561646_m1
HPRT	Rn_01527840_m1

CCL-3: Chemokine (C-C motif) ligand 3, TGF-1ß: Transforming Growth Factor, Il-10: Interleukin 10, MRC-1: Mannose Receptor C-type 1, CXCL-10: C-X-C motif chemokine 10, Il-1ß: Interleukin 1-beta, IGF-1: Insulin-like growth factor 1, TNFα: Tumor necrosis factor, iNOS: Nitric oxide synthases, Il-6: Interleukin 6, HPRT: Hypoxanthine-guanine-phosphoribosyl-transferase 1.

## Abbreviations

DMF	Dimethylfumarate
MMF	Monomethylfuamarate
LPS	Lipopolysaccharides
IL	Interleukin
Brdu	Bromodeoxyuridine
CNS	Central Nervous System
PLL	Poly-l-lysine
PFA	Paraformaldehyde
OPC	Oligodendrocyte Precursor cell
